# LLIN Evaluation in Uganda Project (LLINEUP)–effects of a vector control trial on *Plasmodium* infection prevalence and genotypic markers of insecticide resistance in *Anopheles* vectors from 48 districts of Uganda

**DOI:** 10.1038/s41598-024-65050-z

**Published:** 2024-06-24

**Authors:** Amy Lynd, Samuel Gonahasa, Sarah G. Staedke, Ambrose Oruni, Catherine Maiteki-Sebuguzi, Penelope A. Hancock, Erin Knight, Grant Dorsey, Jimmy Opigo, Adoke Yeka, Agaba Katureebe, Mary Kyohere, Janet Hemingway, Moses R. Kamya, Daniel McDermott, Eric R. Lucas, Martin J. Donnelly

**Affiliations:** 1 Liverpool School of Tropical Medicine, Pembroke Place, Liverpool, L3 5QA UK; 2 Infectious Diseases Research Collaboration, 2C Nakasero Hill Road, Kampala, Uganda; 3 Imperial College, Norfolk Place, London, W2 1PG UK; 4 University of California, San Francisco, San Francisco, CA 94110 USA; 5 Uganda Ministry of Health, Kampala, Uganda; 6 Department of Medicine, Makerere University, Kampala, Uganda

**Keywords:** Malaria, Long-lasting insecticidal nets (LLINs), Piperonyl butoxide (PBO), Uganda, Cluster-randomised trial, Insecticide resistance, Evolutionary genetics, Molecular ecology

## Abstract

Pyrethroid bednets treated with the synergist piperonyl butoxide (PBO) offer the possibility of improved vector control in mosquito populations with metabolic resistance. In 2017–2019, we conducted a large-scale, cluster-randomised trial (LLINEUP) to evaluate long-lasting insecticidal nets (LLINs) treated with a pyrethroid insecticide plus PBO (PBO LLINs), as compared to conventional, pyrethroid-only LLINs across 104 health sub-districts (HSDs) in Uganda. In LLINEUP, and similar trials in Tanzania, PBO LLINs were found to provide greater protection against malaria than conventional LLINs, reducing parasitaemia and vector density. In the LLINEUP trial, we conducted cross-sectional household entomological surveys at baseline and then every 6 months for two years, which we use here to investigate longitudinal changes in mosquito infection rate and genetic markers of resistance. Overall, 5395 female *Anopheles* mosquitoes were collected from 5046 households. The proportion of mosquitoes infected (PCR-positive) with *Plasmodium falciparum* did not change significantly over time, while infection with non-*falciparum* malaria decreased in *An. gambiae* s.s., but not *An. funestus*. The frequency of genetic markers associated with pyrethroid resistance increased significantly over time, but the rate of change was not different between the two LLIN types. The knock-down resistance (*kdr*) mutation *Vgsc*-995S declined over time as *Vgsc*-995F, the alternative resistance mutation at this codon, increased. *Vgsc*-995F appears to be spreading into Uganda. Distribution of LLINs in Uganda was previously found to be associated with reductions in parasite prevalence and vector density, but here we show that the proportion of infective mosquitoes remained stable across both PBO and non-PBO LLINs, suggesting that the potential for transmission persisted. The increased frequency of markers of pyrethroid resistance indicates that LLIN distribution favoured the evolution of resistance within local vectors and highlights the potential benefits of resistance management strategies.

*Trial registration*: This study is registered with ISRCTN, ISRCTN17516395. Registered 14 February 2017, http://www.isrctn.com/ISRCTN17516395.

## Introduction

Long-lasting insecticidal nets (LLINs) are the principal tool for malaria control in Africa and were the major driver of the decline in malaria mortality between 2000 and 2015^1,2^. Growing concerns over the rapid spread of resistance to the pyrethroid insecticides with which the nets aretreated​^3,4^ have been partially assuaged by promising trials of new generation LLINs treated with the synergist piperonyl butoxide (PBO)^5–8^, which inhibits the activity of cytochrome P450s, one of the major causes of insecticide resistance in malaria vectors. The LLIN Evaluation in Uganda Project (LLINEUP, Trial registration: ISRCTN17516395, 14/02/2017), was a cluster randomised trial of conventional LLINs (PermaNet 2.0 and Olyset) and PBO-LLINs (PermaNet 3.0 and Olyset Plus). The trial found that parasite prevalence, adjusted for baseline, was 20% lower in children from communities receiving PBO LLINs than in those receiving conventional LLINs, for up to 25 months post-LLIN distribution (parasite prevalence ratio 0.80 [95% CI 0.69–0.93], *P* = 0.0048)​^5^. Vector abundance, again adjusted for baseline, was 73% lower in PBO communities than in non-PBO communities (Vector density ratio 0.27 [95% CI 0.21–0.36], *P* < 0.0001)​^5^ .

In this paper we report on the frequency of *Plasmodium* infection and molecular markers of insecticide resistance over the course of the trial, testing four hypotheses about the change over time after bednet deployment. First, regarding parasite infection, we tested the following hypothesis:

### H_1_

The previously reported decline in human malaria rates after LLIN distribution leads to a decline in *Plasmodium* infection in *Anopheles* mosquitoes.

Insecticide resistance is widespread in malaria vectors in Uganda​^9–13^, and has been implicated in the limited epidemiological impact of vector control campaigns​^14–16^. The World Health Organization (WHO) has identified insecticide resistance management (IRM) as a key strategy to prolong the effective lifespan of insecticide-based interventions​^17^. A central tenet of IRM programmes is that use of insecticides within the public health sector selects for resistance and therefore the rational deployment of resistance management tools through rotations, mixtures and mosaics etc*.* can delay the onset of resistance. However, it is unclear whether the application of insecticides for public health is the primary driver of insecticide resistance, or whether use of insecticides for agriculture contributes. Demonstrating the role of the former is important for developing IRM strategies. The primary vector of malaria in Uganda is *An. gambiae s.s*^10^, and it is the species for which the largest array of molecular markers are available. We thus focused our analyses on this species.

In a survey of 5,200 households conducted from March to June 2017, in advance of LLIN distribution, only 14.8% of residents lived in a household with adequate LLIN coverage, with nets having been delivered during the previous Universal Coverage Campaign (UCC) between 29 to 53 months earlier and therefore being towards the end of their useful lifespan​^18^. Following the UCC conducted as part of the LLINEUP study, 96.7% of surveyed households had an LLIN with 70.8% having adequate coverage. These values remained far in excess of baseline throughout the trial​^5^. The scale and diverse ecologies over which the LLINEUP trial was conducted afforded us the opportunity to test three hypotheses regarding molecular markers of insecticide resistance in this species. First, distribution of new bednets, with resulting increasing bednet coverage and renewal of the active ingredient concentration, should lead to an increase in pyrethroid resistance. We therefore tested the following hypothesis:

### H_2_

The use of pyrethroid insecticides on LLINs selects for an increase in frequency of molecular markers of pyrethroid resistance over the course of the intervention.

A key assumption of IRM strategies is that the use of insecticide synergists, such as PBO, should retard the evolution of resistance. We examine whether there was any evidence for differences in resistance marker frequency changes between intervention arms receiving conventional LLINs and those receiving PBO-LLINs. A priori we proposed the following hypothesis:

### H_3_

The rate of change in frequency in cytochrome P450 resistance markers will differ in clusters which received PBO-LLINs vs conventional LLINs.

One of the resistance markers we genotype, the *Cyp6aap-Dup1* haplotype, is more strongly associated with resistance to Class II pyrethroids (including deltamethrin and α-cypermethrin) than to Class I pyrethroids (permethrin)​^12^. We therefore propose:

### H_4_

The rate of increase in the *Cyp6aap*-Dup1; ZZB-TE, *Cyp6p4*-236M triple mutant haplotype will be greater in clusters which received conventional deltamethrin LLINs (PermaNet 2.0) relative to clusters which received conventional permethrin LLINs (Olyset).

## Methods

### Household selection, mosquito collection and processing.

**Figure 1:**
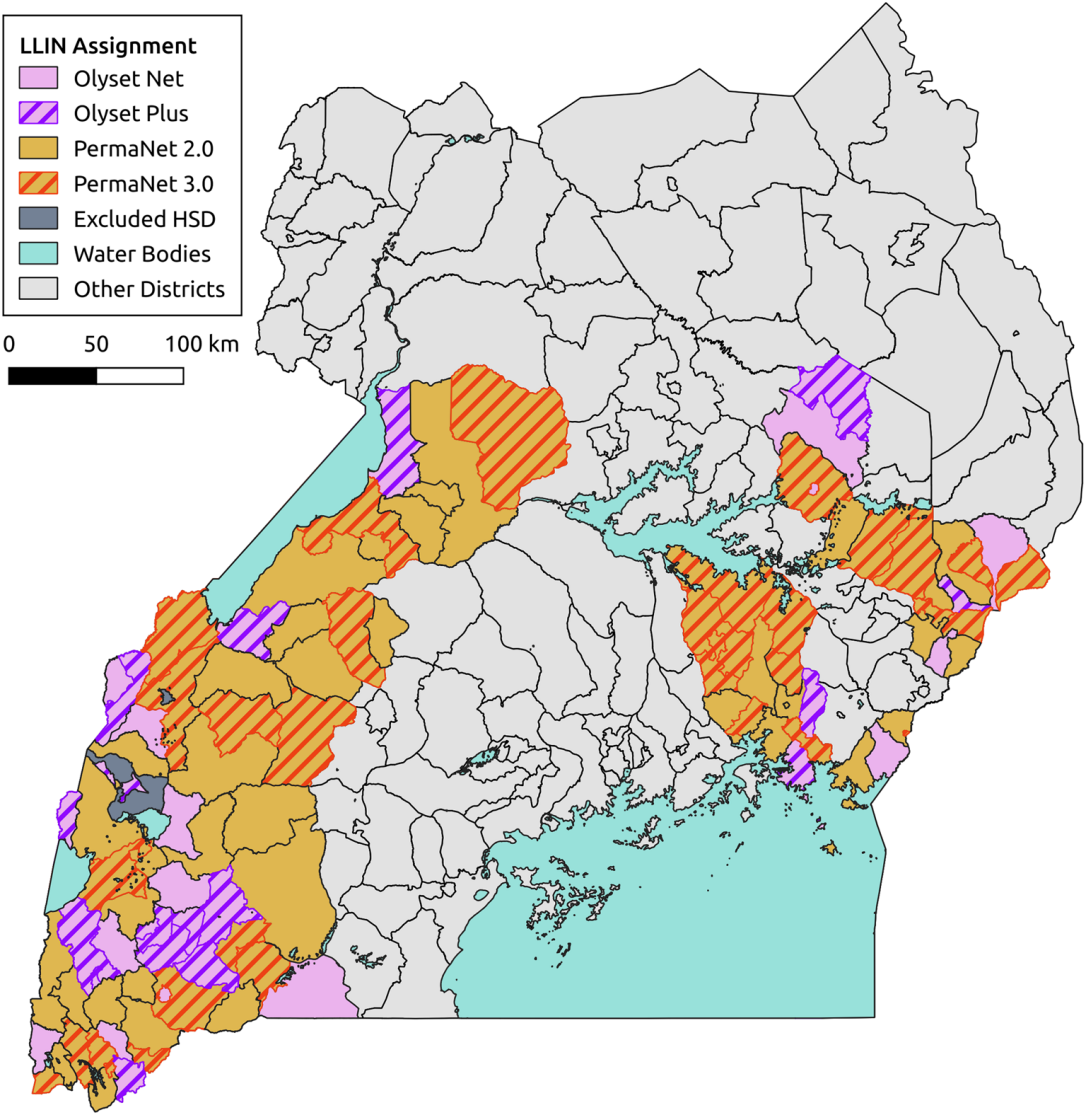
Map of mosquito collection locations within the LLINEUP cluster-randomised control trial. Intervention arm is indicated by colour with the 14 clusters which were omitted during final collection round (25 months post LLIN distribution) indicated with hatched shading. Figure created using QGIS v 3.32 (http://qgis.osgeo.org).

Full details of the sampling procedures are given in​^10,19^, Fig. 1. In brief, in each round of surveys, 50 households were randomly selected from an enumeration list of households in each of the 104 health sub-districts (HSDs) for the cross-sectional community surveys. Of those 50 households, 10 were randomly selected to take part in the entomology surveys, giving a maximum of 1040 households for entomological surveillance. In the final round of surveys (25 months post net distribution) it was only possible to survey 90 of the 104 HSDs due to restrictions resulting from the COVID-19 pandemic. Mosquitoes were collected using Prokopack aspirators​^20^ and DNA extractions were carried out on the head and thorax using Nexttec Biotechnologie DNA extraction plates (Nexttec Biotechnologie GmbH, Hilgertshausen, Germany), and these extractions were used for all subsequent molecular assays. *Anopheles gambiae *s.l. and *An. funestus *s.l. mosquitoes were identified to species level by PCR​^21,22^, with 46 out of 5442 samples failing to amplify and therefore being discarded from further analysis. Malaria infections in *An. gambiae *s.l. and *An. funestus *s.l. were detected by a *P. falciparum*, *P. vivax*, *P. ovale* and *P. malariae* Taqman assay​^23^*.* Since only the head and thorax were used for DNA extraction, rather than the gut, presence of *Plasmodium* is likely to indicate sporozoite infection. Even if there is slight error in our PCR-based estimates, our use of the results is not to obtain an exact sporozoite rate, but rather to compare infection before and after intervention, for which the PCR-based measure is suitable.

The baseline entomological screening identified that *An. gambiae *s.s. was the primary vector across the study area​^10^ and we therefore prioritised molecular work on this species. *An. gambiae *s.s. females were screened for pyrethroid resistance mutations; *Vgsc*-995F, *Vgsc*-995S, *Vgsc*-1570Y, *Cyp6aap*-Dup1 triple mutant haplotype (consisting of *Cyp6aap*-Dup1 itself, *Cyp6p4*-236M and the transposable element insertion ZZB), *Cyp4J5*-43F and *Coeae1d* following standard protocols​^12,24–27^. The 2La chromosome inversion karyotype of *An. gambiae *s.s. specimens was assessed by PCR​^28^.

Data analysis was carried out in R statistical software version 4.1.3​^29^ (www.r-project.org). Analysis of sporozoite infection and molecular marker frequency data used General Linear Models with logit link function for a binomial dependent variable, implemented in the R package glmmTMB​^30^. Collections were conducted over 5 rounds, but mosquito numbers dropped sharply after the LLINs were distributed (Supplementary Fig. 1), as previously reported​^5,7^, with a notable increase by round 5 (Table 1). Thus, few mosquito samples were available for evaluation in intermediate rounds, particularly in communities that received PBO LLINs. We therefore ran the analysis of genotype frequencies both using data from across all rounds, and focusing on comparisons between baseline and Round 5 (Table 3 and​^5^).

**Table 1:** Numbers of female mosquitoes collected from 104 health sub districts across all 5 collection rounds.

	Round 1 (Baseline)	Round 2	Round 3	Round 4	Round 5^b^
*An. gambiae s.s*	1284	191	441	256	815
*An.* arabiensis	80	36	61	117	74
*An. funestus*	432	194	250	719	435
Other Anophelines^a^	1	2	3	1	3

^a^includes *An. coluzzii, An. parensis, An. leesoni, An. rivulorum* and *An. mouchetti.*

^b^Data from only 90 HSDs due to COVID-19 impacts.

For each of our four hypotheses, we identified the model best suited to test the significance of the term of interest. We kept the total number of terms as small as possible, to avoid over-fitting, while still controlling for the main potential confounding factors: “Location”, “Arm” and “HSD”. The terms in these models are: “*Plasmodium*” (binomial variable of presence/absence of *Plasmodium* infection according to PCR), “Marker” (binomial variable of presence/absence of mutant form at the haplotype level), “Round” (intervention round 1–5 coded as numeric variable), “Round(1vs5)” (intervention rounds 1 and 5 only, coded as factor), “Location” (East or West Uganda), “Arm” (PBO or non-PBO (conventional) LLIN as treated), “HSD” (health sub-district).

We tested H_1_ by obtaining the *P* value (*anova* function with chi-squared test in R) associated with removing the “Round” term in the following model: *Plasmodium* ~ Round + Location + Arm + (1|HSD). We tested H_2_ by considering the effect of removing the “Round” term in the following two models: Model 1—Glmm (Marker ~ Round + Location + Arm + (1|HSD)); Model 2—Glmm (Marker ~ Round(1vs5) + Location + Arm + (1|HSD)). The first model takes all five round, while the second compares only round 1 against round 5. Similarly, we tested H_3_ by considering the effect of removing the Round: Arm interaction term in the following two models: Model 3—Glmm (Marker ~ Round + Location + Arm + Round: Arm + (1|HSD)); Model 4—Glmm (Marker ~ Round(1vs5) + Location + Arm + Round(1vs5):Arm + (1|HSD)). Finally, we tested H_4_ by taking the *P* value of the Round: Insecticide interaction term in the following models, including only samples from the conventional nets arm: Model 5—Glmm (Marker ~ Round + Location + Insecticide + Round: Insecticide + (1|HSD)); Model 6—Glmm (Marker ~ Round(1vs5) + Location + Insecticide + Round(1vs5): Insecticide + (1|HSD)). The* P*-values of relevance to our hypotheses were corrected for multiple testing by controlling the false discovery rate (FDR)​^31^.

Spatiotemporal variation in marker frequencies was analysed by fitting a Bayesian geostatistical model to the frequencies of each marker observed in the mosquitoes collected from each household in rounds 1–5​^32^. The numbers of each allele present in each sample were assumed to follow a binomial distribution, with a mean probability modelled as a spatiotemporal random effect depending on latitude, longitude and round. Spatial autocorrelation was modelled using a Gaussian Markov random field and temporal autocorrelation was an autoregressive model of order 1. Models were fitted using the R-INLA package​^33,34^. The script to produce these models and map the outcome are available at https://github.com/vigg-lstm/llineup-genotyping/tree/main/INLA_mapping.

*Anopheles* household abundance data were reported previously in the main trial papers​^5,7^ with molecular data from baseline collections published in the baseline entomology paper​^10^. All data from the 6, 12, 18 and 25 month collection rounds and associated analyses are unique to this manuscript. All collection data and analytical routines are available on GitHub (https://github.com/vigg-lstm/llineup-genotyping).

### Ethics approval and consent to participate

The study was approved by the Ugandan National Council for Science and Technology (UNCST Ref HS 2176), Makerere University School of Medicine Research & Ethics Committee (SOMREC 2016-133), London School of Hygiene & Tropical Medicine Ethics Committee (LSHTM Ref 12019), and the Liverpool School of Tropical Medicine (Ref 16-072), which sponsored the study. All research was performed in accordance with relevant guidelines/regulations. Written informed consent to participate in the study was obtained by the head of household (or their designate) for all participating households.

## Results

Overall, 5395 female *Anopheles* mosquitoes were collected (resting indoor collections using aspirators) from 5046 households in the five surveys (Table 1), including 1797 in round 1 (baseline survey), 423 in round 2 (6-months post-LLIN distribution), 755 in round 3 (12-months), 1093 in round 4 (18-months) and 1327 in round 5 (25-months). At baseline, the prevalence of *Plasmodium falciparum* infection in *An. gambiae* s.s. was 5.6% (72/1284) and in *An. funestus* was 3.5% (15/432). Other *Plasmodium* species (*P. vivax*, *P. ovale*, and *P. malariae*) were detected less commonly in both *An. gambiae* s.s. (1.2%, 16/1284) and *An. funestus* (1.4%, 6/432)​^10^. No *Plasmodium* infections were detected in *An. arabiensis* (Supplementary Table 1) and they were excluded from further analysis.

### H_1_

The previously reported decline in human malaria rates after LLIN distribution leads to a resulting decline in *Plasmodium* infection in *Anopheles* mosquitoes.

**Figure 2:**
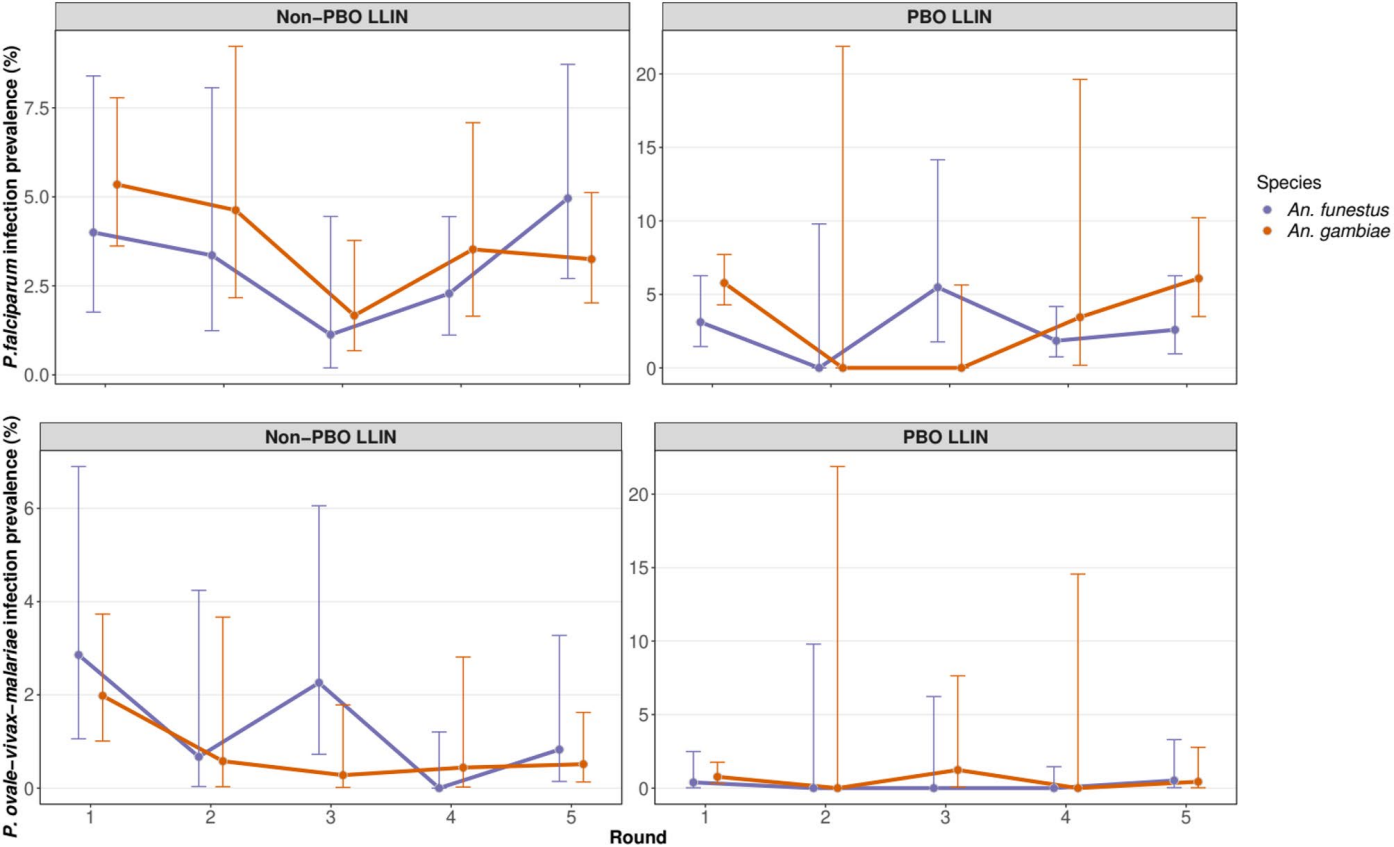
*Plasmodium* infection prevalence in *An. gambiae and An. funestus*. Point prevalence estimate is shown with associated 95%CIs. Data were collected simultaneously but are plotted offset for ease of viewing. Round 1 was the baseline collection with follow up rounds at approximately six-monthly intervals (see text).

The prevalence of *P. falciparum* infection in *Anopheles* mosquitoes did not change significantly over the course of the study (*P* = 0.074 and *P* = 0.86 for *An. gambiae* and *An. funestus* respectively). In contrast, the combined prevalence of other *Plasmodium* species decreased significantly in *An. gambiae* (*P* = 0.018)*,* but not *An. funestus* (*P* = 0.36; Fig. 2; Table 2), although after correction for multiple testing, the false discovery rate was found to be slightly higher than 5% (*Q* = 0.073). There was no evidence for a significant interaction between collection round and study arm (ie, a difference in the slope of infection prevalence over time between intervention arms) for either *P. falciparum* or other non-falciparum *Plasmodium*.

**Table 2:** GLMM analysis of *Plasmodium* infection prevalence in the two main vector species, showing term coefficient; *P*-value (*Q*-value), where *Q* value is the adjusted *P* value by the Benjamini–Hochberg correction.

	*An. gambiae *s.s.^a^	*An. funestus*^a^
Model 1 (Pfal^b^)	− 0.107; *P* = 0.07 (*Q* = 0.15)	0.013; *P* = 0.86 (*Q* = 0.86)
Model 2 (OVM^c^)	− 0.336; ***P* = 0.018** (*Q* = 0.07)	− 0.246; *P* = 0.36 (*Q* = 0.48)

Significant unadjusted* P*-values shown in bold. Model 1- Glmm (*P. falciparum* ~ Round + Location + Arm + (1|HSD)); Model 2- Glmm (*P. OVM* ~ Round + Location + Arm + (1|HSD)). For both models a Round:Arm interaction term was also included but was not found to improve model fit.

^a^Model coefficient and significance value.

^b^Pfal = *P. falciparum.*

^c^OVM = *P. ovale*, *P. vivax* and *P. malariae.*

The resistance associated variant *Vgsc*-1570Y was not found in any specimen and is not discussed further. There was no significant association between any of the genotypic markers screened and infection with *P. falciparum* or the other non-falciparum *Plasmodium* (Supplementary Fig. 2).

### H_2_

The use of pyrethroid insecticides on LLINs selects for an increase in frequency of molecular markers of pyrethroid resistance over the course of the intervention.

**Figure 3:**
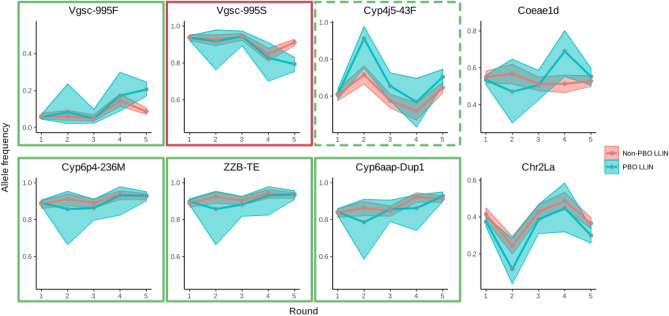
Insecticide resistance marker prevalence *in Anopheles gambiae *s.s. across the baseline and four post LLIN distribution collection rounds. Markers in green external boxes show significant increases (see Table 3) over the course of the trial, red boxes indicate significant decreases (Table 3). Dashed line indicates significant change observed only when GLMM included all five collection points.

Five pyrethroid resistance markers showed a significant change in frequency (*Vgsc*-995F; *Vgsc*-995S; *Cyp6aap*-Dup1; ZZB-TE, *Cyp6p4*-236 M) (Fig. 3 and Table 3), either over the course of study follow-up (*P* = 1 × 10^–9^, *P* = 3 × 10^–9^, *P* = 9 × 10^–12^, *P* = 4 × 10^–5^, *P* = 7 × 10^–5^, for the five markers respectively; Table 3 Model 1), or when comparing frequencies at baseline with those from the final collection round (*P* = 3 × 10^–9^, *P* = 1 × 10^–8/^, *P* = 7 × 10^–12^, *P* = 3 × 10^–6^, *P* = 9 × 10^–6^; Table 3 Model 2). A sixth marker (*Cyp4j5*-43F) also showed significant changes by raw *P-*values over the course of the study (*P* = 0.03), but the *Q* values after FDR correction were around 0.06, indicating that accepting these as significant would lead to an overall rate of 6% false positives among our significant *P*-values. The statistical significance of the change for *Cyp4j5-*43F is therefore ambiguous. *Cyp6aap*-Dup1; ZZB-TE, *Cyp6p4*-236 M are in near full linkage disequilibrium on a triple-mutant haplotype​^12^, so subsequent analyses focused on *Cyp6p4*-236 M as representative of this haplotype. No significant changes in frequency for 2La or *Coeae1d* resistance markers were observed (respectively, *P* = 0.1 and *P* = 0.63 over the course of the study, *P* = 0.37 and *P* = 0.75 when comparing rounds 1 and 5). In the 25 months following LLIN distribution, all resistance markers increased significantly in frequency, except for *Vgsc*-995S which decreased significantly (dropping from 94% in both conventional and PBO-LLINs to 79% (PBO) and 91% (conventional) frequency). These increases are consistent with the hypothesis that LLINs treated with pyrethroids (a public health intervention) exert selective pressure and drive pyrethroid resistance. The *Vgsc-995S* mutation is in negative linkage with *Vgsc*-995F, with relatively few wild-type alleles in the population​^10^. Thus, the decrease in *Vgsc*-995S is an expected consequence of the increase in *Vgsc*-995F.

**Table 3:** Summary of GLMM analyses of resistance allele frequency changes in *Anopheles gambiae s.s*, showing term coefficient, *P*-value (*Q*-value), where *Q* value is the adjusted *P* value by the Benjamini–Hochberg correction.

Hypo-thesis	Model	Data included	Variable tested	*Vgsc*-995F^b^	*Vgsc*-995S^b^	*Cyp4j5*-43F^b^	*Cyp6p4*-236 Mb^,c^
1	Model 1	All	Round	0.20; ***P* = 1 × 10**^**–9**^ (*Q* = 2 × 10^–8^)	− 0.19; ***P* = 3 × 10**^**–9**^ (*Q* = 2 × 10^–8^)	0.04; ***P* = 0.03** (*Q* = 0.06)	0.12; ***P* = 7 × 10**^**–5**^ (*Q* = 2 × 10^–4^)
	Model 2	All	Round^a^	0.87; ***P* = 3 × 10**^**–9**^ (*Q* = 2 × 10^–8^)	− 0.82; ***P* = 1 × 10**^**–8**^ (*Q* = 5 × 10^–8^)	0.12; *P* = 0.22 (*Q* = 0.36)	0.54; ***P* = 9 × 10**^**–6**^ (*Q* = 3 × 10^–5^)
2	Model 3	All	Interaction (Arm:Round)	ND	ND	0.09; ***P* = 0.025** (*Q* = 0.056)	− 0.07; *P* = 0.24 (*Q* = 0.36)
	Model 4	All	Interaction (Arm:Round)^a^	ND	ND	0.48; ***P* = 0.014** (*Q* = 0.036)	− 0.29; *P* = 0.27 (*Q* = 0.37)
3	Model 5	Only Non-PBO LLIN	Interaction (Insecticide: Round)	ND	ND	ND	− 0.05; *P* = 0.55 (*Q* = 0.62)
	Model 6	Only Non-PBO LLIN	Interaction (Insecticide: Round)^a^	ND	ND	ND	− 0.31; *P* = 0.34 (*Q* = 0.44)

Significant unadjusted* P*-values shown in bold.

^a^Model compares marker frequency data from baseline and data from 25 months.

^b^Model coefficient and significance value.

^c^Used as the sole marker for the triple mutant haplotype as this marker is in linkage disequilibrium with ZZB-TE and *Cyp6aap*-Dup1. In these collections. *Coeae1d and Chr2La not included as not significant in models 1 and 2 (not relevant for other comparisons) Model 1—Glmm (Marker ~ Round + Location + Arm + (1|HSD)); Model 2—Glmm (Marker ~ Round(1vs5) + Location + Arm + (1|HSD)); Model 3—Glmm (Marker ~ Round + Location + Arm + Round:Arm + (1|HSD)); Model 4—Glmm (Marker ~ Round(1vs5) + Location + Arm + Round:Arm + (1|HSD)); Model 5(Non-PBO-LLINs)—Glmm (Marker ~ Round + Location + Insecticide + Round:Insecticide + (1|HSD)); Model 6(Non-PBO-LLINs)—Glmm (Marker ~ Round(1vs5) + Location + Insecticide + Round:Insecticide + (1|HSD)). The “Arm” term distinguishes conventional LLINs and PBO-LLINs but not between deltamethrin and permethrin-treated nets. The “Insecticide” term distinguishes between deltamethrin and permethrin-treated nets. ND = Not done.

**Figure 4:**
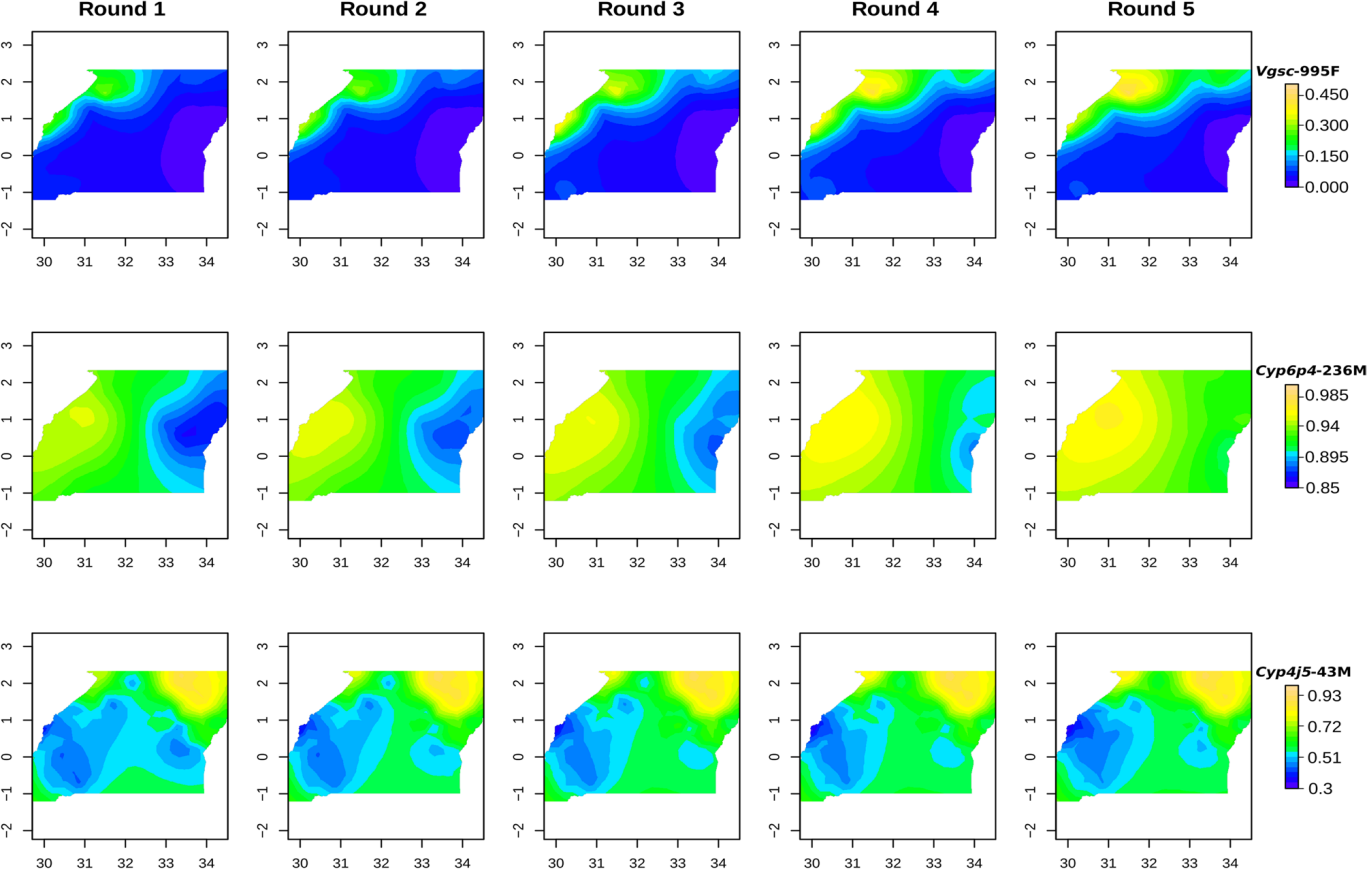
Mapping of mutant allele frequencies over collection rounds for *Vgsc-*995F, *Cyp6p4*-236 M and *Cyp4j5*-43F. X and Y axis tickmarks show longitude and latitude respectively. Note colour scales do not carry over across rows.

Mapping the change in frequency of *Vgsc-*995F suggested that this mutation gradually spread from the North-West of the country over the 2-year study period (Fig. 4). In contrast, the increase in frequency of *Cyp4j5*-43F occurred in all areas, and particularly in the Southern regions. At baseline, the *Cyp6p4*-236M (triple-mutant haplotype) was already present at high frequency (> 65%) across the study area, and the greatest increases in frequency of this allele were in the North-East, where the baseline frequencies were lowest.

### H_3_

The rate of change in frequency of cytochrome P450 resistance markers will differ in clusters which received PBO-LLINs vs conventional LLINs.

*Cyp4j5*-43F and *Cyp6p4*-236M (triple-mutant haplotype) showed contrasting trends in frequency (Fig. 3). For *Cyp4j5-*43F, the interaction between collection round and LLIN arm was significant by raw *P*-value, with false discovery rates close to 0.05 both over the course of the study (*P* = 0.025, *Q* = 0.056, Table 3 Model 3) and when comparing pre-LLIN distribution frequencies with the final collection round (*P* = 0.014, *Q* = 0.036, Table 3 Model 4). The direction of the interaction indicates that the rate of increase in this P450-mediated resistance mechanism is, perhaps counter-intuitively, higher in clusters which received PBO-LLINs. Conversely there was no evidence for a significant interaction between round and intervention arm for the triple haplotype marker, *Cyp6p4*-236M.

### H_4_

The rate of increase in the triple mutant haplotype will be greater in clusters which receive deltamethrin LLINs (PermaNet 2.0) relative to clusters which receive permethrin LLINs (Olyset).

There was no evidence that rates of change in frequency of the triple-mutant haplotype were different between clusters receiving conventional LLINs treated with either permethrin or deltamethrin (*P* = 0.55 over the course of the study, *P* = 0.34 between rounds 1 and 5; Table 3 models 5 and 6).

## Discussion

To investigate the impact of LLINs on the emergence and spread of insecticide resistance in Uganda, we evaluated *Anopheles* mosquitoes collected from 48 districts over the 2-year follow-up period of the LLINEUP trial. We found that although parasite prevalence and vector density decreased in both study arms following the distribution of LLINs​^5^, the rate of infection with *Plasmodium falciparum* in sampled mosquitoes did not change significantly over the study, while rates of infection with other *Plasmodium* species decreased significantly in *An. gambiae,* but not *An. funestus*. Since the number of mosquitoes collected following LLIN distribution was low, it is possible that the lack of significance is a result of low statistical power. However, in the case of *An. funestus,* there was no suggestion of an overall decrease in infection over time (the model coefficient was positive). The reduction in parasite prevalence​^5^ and mosquito numbers does not appear to have markedly reduced infection rates in the *Anopheles* vectors analysed. In the LLINEUP trial, the reduction in parasite prevalence was observed in children aged 2–10 years. It is possible that parasitaemia in older children and adults persisted, thus providing opportunities for mosquitoes to take infected blood meals from this reservoir of older residents. Increased mosquito mortality, inferred from reduced vector collections, is expected to result in a younger mosquito population, leading to a smaller proportion of mosquitoes living long enough to become infective. However, increased adult mortality does not necessarily result in a younger age distribution if it causes a population decline​^35^, and mosquito infection rate may therefore be unaffected.

We found no evidence of association between resistance marker genotype and infection status. These results contrast to those from a previous study which detected an association between *Vgsc*-995S genotype and infection, consistent with the hypothesis that mosquitoes carrying resistant alleles had increased longevity and were therefore more likely to survive the parasite extrinsic incubation period​^36^. One difference that could explain these results is that overall *Vgsc*-995 and *Cyp6p4*-236M mutant frequencies were high in our study, with very few wild-type alleles found in the population. There may therefore have been too few fully susceptible individuals to detect an effect of resistance on *Plasmodium* infection.

There was clear evidence of increases in genotypic markers of pyrethroid resistance over the study. Although it not possible to randomise resistance between trial arms, and thus we cannot exclude the possibility that marker frequency would have increased even in the absence of LLINs, we consider the considerable scale-up of LLIN coverage to be the most likely explanation for the results as the mosquitoes were collected from HSDs representing ≈40% of the surface area of Uganda, encompassing marked differences in ecology, altitude, socio-economic status of communities etc.^5^. Previous work to correlate LLIN distributions with changes in resistance have yielded contrasting results, arguably due to their reliance upon inherently noisy resistance phenotyping approaches​^37^. Our use of genotypic markers provides a metric that can be accurately quantified, reducing the noise in the statistical analysis. The significant increase in *Vgsc*-995F frequency at the expense of the alternative *Vgsc-*995S allele suggests that the former mutation is gradually replacing the latter, and mapping of allele frequencies indicates that this replacement is centred in the North-West of the country. *Vgsc*-995F is a predominantly West- and Central-African allele, and thus its higher frequency in the North-Western part of Uganda is consistent with a gradual spread eastwards. *The Vgsc-*995F was first observed in *An. gambiae *s.s. from the region in 2012​^38^. At this time the *Vgsc*-995S variant was near fixation​^39^ and analyses suggested that the *Vgsc*-995S mutation was older​^40^ and more strongly associated with DDT rather than pyrethroid resistance​^41,42^. The increase in frequency of the *Vgsc-*995F mutation provides additional support to our contention that LLINs are a major driver of pyrethroid resistance.

PBO LLINs have been promoted as an intervention that can overcome, at least in part, cytochrome P450-mediated resistance. A priori we would have predicted that mosquito populations sampled from communities that received PBO LLINs would show a decrease in P450-resistance markers relative to conventional LLINs. However whilst there were contrasting patterns observed between different P450 marker systems, the only evidence of a significant change was a relative increase in frequency of the *Cyp4j5*-43F marker. It may be that incomplete suppression of cytochrome P450s does not remove the selective pressure to upregulate this form of resistance, but rather escalates an arms race in which mosquitoes further upregulate cytochrome P450-mediated resistance to overcome the suppressive effects of PBO.

## Conclusions

The large-scale deployment of LLINs in Uganda in 2017–2019 was associated with an increase in the frequency of genotypic markers of pyrethroid resistance, but not in the frequency of *P. falciparum* infection in mosquitoes. The reduction in malaria prevalence resulting from the LLIN distribution campaign was therefore likely the result of decreased mosquito numbers, rather than fewer infective mosquitoes. The increase in resistance allele frequency suggests that public health interventions, such as LLINs, can apply selective pressure which drives the evolution of insecticide resistance, supporting the need for resistance management strategies.

### Supplementary Information

Supplementary Information.

## Data Availability

The datasets reported herein are publicly available in the GitHub repository https://github.com/vigg-lstm/llineup-genotyping, as are the scripts used in the analysis of these data. GPS coordinates of the households used in the study have been removed for data protection. Benefits Generated: A research collaboration was developed with scientists from the country providing genetic samples, all collaborators are included as co-authors, the results of research have been shared with the provider communities and the broader scientific community, and the research addresses a priority concern, in this case the impact of PBO-LLINs on the evolution of resistance to insecticides in malaria mosquitoes.
